# Naturalistic Associations Between Childhood Maltreatment, Compulsivity, and Eating Disorder Symptoms Over a 12‐Months Period Among Individuals With Anorexia Nervosa

**DOI:** 10.1002/erv.70041

**Published:** 2025-10-13

**Authors:** Elizabeth N. Dougherty, Glen Forester, Matthew F. Murray, Brianne N. Richson, Angeline R. Bottera, Samantha E. Weiss, Jennifer E. Wildes

**Affiliations:** ^1^ Department of Psychiatry and Behavioral Neuroscience University of Chicago Chicago Illinois USA; ^2^ Center for Biobehavioral Research Sanford Research Sioux Falls South Dakota USA; ^3^ Department of Psychiatry and Behavioral Science School of Medicine and Health Sciences University of North Dakota Grand Forks North Dakota USA; ^4^ Department of Psychology University of Kansas Lawrence Kansas USA

**Keywords:** anorexia nervosa, childhood maltreatment, compulsivity, eating disorder symptoms

## Abstract

**Introduction:**

Childhood maltreatment is associated with more severe eating disorder (ED) symptoms and compulsivity, and compulsivity is implicated in anorexia nervosa (AN). However, the role of childhood maltreatment and compulsivity in shaping the clinical course of AN remains unclear.

**Objective:**

We investigated whether childhood maltreatment was associated with compulsivity, and whether childhood maltreatment and compulsivity moderated changes in ED symptoms over time following discharge from treatment in AN.

**Method:**

Individuals (*N* = 194; Age = 16–62) with AN restricting type (AN‐R) and AN binge‐eating/purging type (AN‐BP) completed a diagnostic interview and self‐report measures of compulsivity and childhood maltreatment upon admission to treatment. They completed follow‐up assessments of ED symptoms at discharge and three, six, and 12 months post‐discharge.

**Results:**

Higher exposure to childhood maltreatment was associated with greater compulsivity at admission in individuals with AN‐BP. Childhood maltreatment moderated changes in ED symptoms from discharge to 12 months post‐discharge in individuals with AN‐R, such that those with lower levels of maltreatment showed symptom improvement, whereas those with higher levels of maltreatment showed no change in symptoms. Compulsivity did not moderate changes in symptoms.

**Conclusions:**

Findings highlight childhood maltreatment as a potentially important factor to account for in improved interventions for AN.

## Highlights

- Childhood maltreatment was associated with greater compulsivity at admission in individuals with AN‐BP.
- Childhood maltreatment moderated changes in eating disorder symptoms over the 12‐month period following discharge from treatment in individuals with AN‐R.
- Compulsivity did not moderate changes in eating disorder symptoms post‐discharge in individuals with AN‐R or AN‐BP.

## 1 Introduction

Anorexia nervosa (AN) is a debilitating psychiatric disorder characterised by dietary restriction leading to significantly low body weight, perceptual distortions of body weight and shape, and fear of weight gain (American Psychiatric Association 2022). AN is associated with multiple medical complications and significantly higher mortality rates than in non‐eating disorder controls (Auger et al. 2021). The clinical course of AN is often protracted, with symptoms that persist or recur following treatment, particularly within the first year after discharge (Berends et al. 2018; Fichter et al. 2017; Steinhausen 2002). Despite its clinical significance, little is known about underlying factors that may help explain the persistent and severe nature of AN. Further, empirically supported treatments show only low‐to‐modest efficacy (Monteleone et al. 2022), underscoring an urgent need for identification of mechanisms underlying AN that could inform more efficacious interventions.

Childhood maltreatment (e.g., childhood sexual abuse, emotional neglect) is one factor that has been implicated in the aetiology and maintenance of eating disorders, including AN (Convertino and Mendoza 2023; Molendijk et al. 2017). Research suggests that childhood maltreatment is significantly more prevalent among individuals with eating disorders compared to non‐eating disorder controls (Molendijk et al. 2017). Moreover, childhood maltreatment is associated with an earlier age of eating disorder onset and a greater severity of eating disorder symptoms in treatment‐seeking and non‐treatment‐seeking samples (Hazzard et al. 2019; Molendijk et al. 2017; Rossi et al. 2024). Notably, previous research investigating how maltreatment relates to the clinical course of eating disorder symptoms in treatment‐seeking samples has generated mixed findings (Convertino and Mendoza 2023; Day et al. 2024; Liebman et al. 2025). For instance, some studies have found no significant difference in eating disorder treatment outcomes at the end of treatment between individuals with and without histories of maltreatment (Convertino and Mendoza 2023; Liebman et al. 2025). In contrast, other studies have shown that maltreatment is associated with a less favourable course of symptoms post‐treatment, including higher rates of relapse and hospitalisation (Convertino and Mendoza 2023; Day et al. 2024; Liebman et al. 2025). A more consistent finding across studies is that childhood maltreatment is associated with an increased risk of premature treatment termination (Convertino and Mendoza 2023; Day et al. 2024). Findings for AN have been particularly inconsistent, with some evidence suggesting that childhood maltreatment may be more relevant to the binge‐eating/purging subtype of AN (AN‐BP) than the restricting subtype (AN‐R; Convertino and Mendoza 2023; Day et al. 2024; Molendijk et al. 2017). For example, some research indicates that childhood maltreatment is more detrimental to treatment outcomes for AN‐BP than AN‐R (Carter et al. 2006). Further, AN‐BP is more frequently linked to dissociative symptoms and emotion dysregulation (Palmisano et al. 2018; Rowsell et al. 2016) – two factors that have been shown to mediate the relation between maltreatment and treatment outcomes in individuals with eating disorders (Cassioli et al. 2022; Liebman et al. 2025). Given inconsistencies in previous literature, particularly regarding how maltreatment may impact the clinical course of AN, further research on this is warranted.

Childhood maltreatment has also been linked to compulsivity — a trait that promotes rigid, repetitive, and context‐inappropriate behaviour that is disconnected from one's goals (Gordon et al. 2020; Luigjes et al. 2019). Evidence suggests that childhood maltreatment may promote compulsivity (Den Ouden et al. 2022; Gordon et al. 2020; Zhou et al. 2020), and compulsivity may contribute to the maintenance of AN (Godier and Park 2014). Supporting the association between childhood maltreatment and compulsivity, studies have shown that individuals who experience childhood maltreatment (e.g., childhood abuse) display more rigid, context‐inappropriate responses during laboratory tasks (Franco and Knowlton 2023; Gordon et al. 2020; Pițur and Miu 2020) and more rigid, automatic behavioural patterns in daily life (Zhou et al. 2020). Similarly, animal research has shown that exposure to prepubertal stress produces compulsive‐like behaviour in female rodents (Brydges et al. 2015). For a child experiencing maltreatment, engagement in rigid, repetitive behaviour may alleviate distress in the short‐term by establishing a sense of predictability and controllability but become maladaptive over time, thereby increasing vulnerability to psychopathology (Tonna et al. 2019). In support of this notion, research has shown that exposure to acute stress promotes rigid behaviour in young children (Seehagen et al. 2015). Moreover, early‐life stress is associated with alterations in frontal‐limbic neurocircuitry that are theorised to underpin compulsive behaviour in multiple psychiatric disorders (Cohodes et al. 2021; Robbins et al. 2024). Studies have also demonstrated an association between compulsivity and AN. For example, research has shown that individuals with AN have higher compulsivity compared to non‐eating disorder controls (Kay et al. 1992; Lloyd et al. 2020). Further, studies have linked compulsivity to more severe eating disorder symptoms in individuals with AN (Lavender et al. 2017; Sternheim et al. 2022).

While previous research supports the relevance of compulsivity to AN, previous studies have relied heavily on cross‐sectional designs; thus, whether compulsivity plays a role in the longitudinal course of AN remains unclear. Further, despite evidence that childhood maltreatment is associated with compulsivity and eating disorder symptoms (Franco and Knowlton 2023; Gordon et al. 2020; Molendijk et al. 2017), no research has investigated the link between childhood maltreatment and compulsivity in individuals with AN. Clarifying naturalistic associations among childhood maltreatment, compulsivity, and eating disorder symptoms in AN following treatment discharge–a period marked by heighted risk of symptom worsening or persistence– may inform the development of interventions aimed at improving long‐term outcomes for AN (Berends et al. 2018; Fichter et al. 2017). Additionally, given that the relation between childhood maltreatment and eating disorder symptoms may differ by AN subtype (Molendijk et al. 2017), exploring these associations separately in AN‐R and AN‐BP may provide a more nuanced understanding of how maltreatment relates to compulsivity and post‐treatment trajectories of eating disorder symptoms in AN.

This study aimed to: (a) investigate whether childhood maltreatment is associated with compulsivity among individuals with AN admitted to intensive eating disorder treatment, and (b) explore whether trajectories of eating disorder symptoms over the 12 months following treatment discharge would vary according to childhood maltreatment and compulsivity. We hypothesised that retrospectively reported childhood maltreatment would be associated with higher compulsivity at treatment admission. We also hypothesised that childhood maltreatment and compulsivity at admission would moderate changes in eating disorder symptoms from discharge to 12 months post‐discharge, such that individuals with higher compulsivity and exposure to maltreatment would display poorer eating disorder symptom trajectories during this period.

## 2 Method

### 2.1 Participants

Participants were 194 individuals with AN admitted to day hospital and inpatient programs at an academic medical centre. Data for this study were collected between 2008 and 2012. Study inclusionary criteria were: (a) a diagnosis of AN, (b) age 16 years or older, (c) a body mass index (BMI) < 18.5 or BMI percentile < 10% (for participants aged 16–19 years) at admission, and (d) current medical stability. Participants had to meet *Diagnostic and Statistical Manual of Mental Disorders*, *fourth edition* (DSM‐IV; APA 1994), criteria for AN, excluding the amenorrhoea criterion. Participants who denied fear of fatness and had a BMI < 17.5 were included, in accordance with descriptions of non‐fat phobic AN (Wildes et al. 2013). Exclusionary criteria were: (a) current psychosis or (b) current intellectual developmental disability. At admission, 84 (43.3%) participants met diagnostic criteria for AN restricting type and 110 (56.7%) met criteria for AN binge‐eating/purging type. Participants were aged 16–62 years (*M* = 26.5, SD = 10.1) and had a mean BMI of 15.7 (SD = 1.8) at admission. The majority identified their biological sex as female (*n* = 185; 95.4%) and their race and ethnicity as non‐Hispanic white (*n* = 185; 95.4%). Additional sociodemographic characteristics of the sample are presented in Table S1.

### 2.2 Procedure

Study procedures were approved by the University of Pittsburgh Institutional Review Board. All participants provided written informed consent prior to participating in the study. For participants under the age of 18 years old, a guardian provided written informed consent, and the participant provided assent. Participants completed an in‐person baseline assessment within 2 weeks of their admission to treatment. During this assessment, they completed clinical interviews assessing psychiatric diagnoses and eating disorder symptoms and completed questionnaires assessing childhood maltreatment and compulsivity. Participants completed follow‐up assessments at discharge and three, six, and 12 months post‐discharge. Follow‐up assessments were conducted either in‐person or by mail or telephone to maximise retention. During follow‐up assessments, participants were readministered a clinical interview to assess eating disorder symptoms. Assessment procedures were performed by bachelor's‐, master's‐ or doctoral‐level research staff who were supervised by licenced clinical psychologists. Of the 194 participants included in this study, 168 (87%) completed discharge assessments, 163 (84.0%) completed three‐month follow‐up assessments, 160 (82.5%) completed six‐month follow‐up assessments, and 154 (79.4%) completed 12‐month follow‐up assessments. More than 90% of participants completed one or more post‐discharge assessments.

### 2.3 Measures

#### 2.3.1 Demographic Variables

A demographic form was used to assess sociodemographic variables. Height and weight were measured objectively at treatment admission and discharge and obtained from participants' medical records. Information about height and weight was used to calculate BMI (kg/m^2^) at admission and discharge.

#### 2.3.2 Co‐Morbid Psychopathology

The Structured Clinical Interview for *DSM‐IV‐TR* Axis I Disorders (First et al. 2007) was used to establish eating disorder and co‐morbid psychiatric diagnoses and assess age of AN onset. This measure has demonstrated good to excellent inter‐rater reliability in previous samples (Lobbestael et al. 2011) and the current sample (Wildes et al. 2013). The Beck Depression Inventory‐Second Edition (BDI‐II) was used to assess depressive symptoms at admission and discharge (Beck et al. 1996). The BDI‐II showed good reliability in the current sample (Cronbach's alpha values ≥ 0.92).

#### 2.3.3 Eating Disorder Symptoms

The Eating Disorder Examination Interview, 16th Edition (EDE; Fairburn et al. 2008) was used to assess the severity of eating disorder symptoms at discharge and three, six, and 12 months post‐discharge. The EDE is a clinician administered semi‐structured interview that comprises four subscales: Dietary Restraint, Eating Concern, Weight Concern, and Shape Concern. A global score is calculated by averaging subscale scores, with higher scores reflecting more severe eating disorder symptoms. The EDE has shown good validity and reliability in previous research (Cooper et al. 1989). Moreover, the EDE scales showed good internal consistencies (Cronbach's alpha range = 0.65 −0.94) in the current sample.

#### 2.3.4 Childhood Maltreatment

The Childhood Trauma Questionnaire‐ Short Form (CTQ‐SF; Bernstein et al. 2003) was used to retrospectively assess childhood maltreatment at admission. The CTQ‐SF is a 28‐item self‐report measure that assesses the severity of five types of childhood maltreatment (i.e., emotional abuse, sexual abuse, physical abuse, emotional neglect, physical neglect). Items on this measure are rated on a 5‐point scale (1 = ‘never true’; 5 = ‘very often true’). Responses were summed to obtain a total score, with a higher score representing more severe childhood maltreatment (across all types of maltreatment). The CTQ‐SF has shown good psychometric properties in previous samples (Bernstein et al. 2003) and good reliability in the current sample (Cronbach's alpha = 0.94).

#### 2.3.5 Compulsivity

The repetition and automation subscale of the Obsessive Compulsive Spectrum Self‐Report questionnaire (OBS‐SR; Dell’Osso et al. 2000; Dell'Osso et al. 2002) was used to assess compulsivity at admission. The OBS‐SR is a self‐report questionnaire that assesses obsessive compulsive psychopathology and related clinical features and temperamental traits. The repetition and automation subscale consists of 12 items that assess the extent to which a person feels compelled to repeat context‐inappropriate behaviours. Items on this measure are rated as either ‘yes’ or ‘no’. A total score is calculated by summing the number of ‘yes’ responses to these items. The OBS‐SR has shown good reliability and convergent validity in previous samples (Dell'Osso et al. 2002; Kang and Kim 2024). In addition, the OBS‐SR showed good reliability in the current sample (Cronbach's alpha = 0.82).

### 2.4 Statistical Analyses

Statistical analyses were performed using R version 4.3.3 (R Core Team). A natural log transformation was performed on multiple variables (i.e., AN illness duration, childhood maltreatment, age, duration of intensive treatment) to correct for positive skew and leptokurtosis. Statistical analyses were conducted separately for AN‐R and AN‐BP. Linear regression analysis with robust standard errors was used to investigate whether childhood maltreatment was associated with higher compulsivity at treatment admission. Preliminary regression analyses were conducted to determine whether the final models should control for depressive symptoms at admission, BMI at admission, age at admission, or AN illness duration. These covariates were chosen based on their theoretical relevance to childhood maltreatment and compulsivity (e.g., Davis et al. 2020; Humphreys et al. 2020). Variables that were significantly associated with compulsivity (i.e., depressive symptoms and AN illness duration) were included as covariates in the final regression models.

Multilevel modelling was used to investigate whether childhood maltreatment and compulsivity at treatment admission moderated changes in eating disorder symptoms from discharge to 12 months post‐discharge, such that individuals with higher compulsivity and exposure to maltreatment would display poorer eating disorder symptom trajectories during this period. Models were estimated using maximum likelihood, which is capable of handling missing data under the assumption that data are missing at random (Li and Stuart 2019). Preliminary analyses were conducted to determine whether models should include non‐linear time effects. Specifically, we examined whether inclusion of quadratic time effects significantly improved model fit compared to a linear time model. The inclusion of quadratic time effects did not significantly improve model fit; therefore, they were excluded from the final models. Each multilevel model included a main effect of time and a between‐person variable (i.e., childhood maltreatment or compulsivity) and their interaction with each other. The outcome variable in these models was global eating disorder symptoms (measured via the EDE global score) from discharge to 12 months post‐discharge. The intercept and time were modelled as random effects. Significant interactions were probed using simple slopes analysis. Preliminary multilevel modelling analyses were conducted to determine whether the final models should control for age at admission, depressive symptoms at discharge, BMI at discharge, AN illness duration, psychiatric co‐morbidity (yes/no), duration of intensive treatment, level of care at admission, or receipt of intensive treatment during the 12‐month follow‐up period. These covariates were selected based on their theoretical relevance to childhood maltreatment, compulsivity and eating disorder symptoms (e.g., Fichter et al. 2017; Humphreys et al. 2020). Variables that were significantly associated with eating disorder symptoms (i.e., co‐morbidity, AN illness duration, age, duration of intensive treatment, receipt of intensive treatment, and depressive symptoms) were retained as covariates in the final multilevel models. For all analyses, alpha was set at 0.05.

## 3 Results

Results of the regression analyses investigating childhood maltreatment and compulsivity at treatment admission are presented in Table 1. Among individuals with AN‐BP, the overall linear regression model was significant (*F*(2, 104) = 14.24, *p* = < 0.001). More severe childhood maltreatment was associated with higher compulsivity. Among individuals with AN‐R, the overall linear regression model was significant (*F*(3, 78) = 6.58, *p* = 0.001); however, childhood maltreatment was not related to compulsivity.

**TABLE 1:** Results of the linear regression analyses examining childhood maltreatment as a predictor of compulsivity at admission.

	*B*	SE	*t*	*p*	95% CI	
Model 1: AN‐R						
Intercept	−2.51	2.91	−0.86	0.390	−8.301	3.273
**Depressive symptoms**	**0.09**	**0.03**	**3.42**	**0.001**	**0.037**	**0.140**
AN illness duration^a^	0.21	0.73	0.28	0.777	−1.240	1.652
Childhood maltreatment^a^	0.61	0.98	0.62	0.535	−1.341	2.562
*R* ^*2*^	0.20					
*F*	6.58					
Model 2: AN‐BP						
Intercept	−0.86	0.75	−1.15	0.254	−2.358	0.629
**Depressive symptoms**	**0.06**	**0.02**	**2.93**	**0.004**	**0.021**	**0.107**
**Childhood maltreatment**	**0.06**	**0.02**	**3.15**	**0.002**	**0.021**	**0.091**
*R* ^*2*^	0.22					
*F*	14.24					

*Note:* Bolded values are significant.

Abbreviations: AN‐BP = Anorexia Nervosa binge‐eating/purging type; AN‐R = AN restricting type; SE = standard error.

^a^ Log transformed variable was used.

**FIGURE 1:**
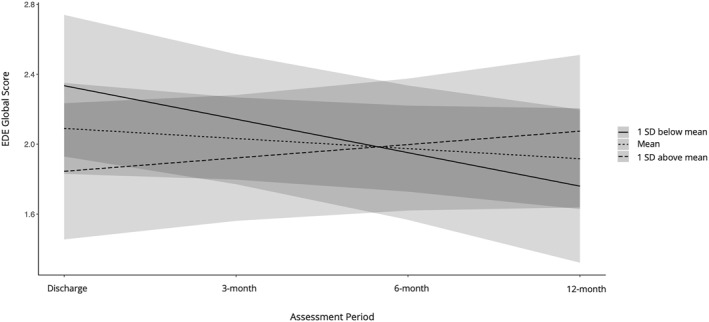
Interaction between childhood maltreatment and change in eating disorder symptoms from discharge to 12‐months post‐discharge in individuals with AN‐R.

Table 2 presents descriptive statistics for global eating disorder symptoms at each timepoint. The results of the multilevel modelling analyses investigating childhood maltreatment and changes in eating disorder symptoms over the 12‐month period following discharge are presented in Table 3. Among individuals with AN‐R, there was a significant interaction between childhood maltreatment and time, suggesting that the linear change in eating disorder symptoms from discharge to 12‐month follow‐up differed depending on the level of childhood maltreatment (Figure 1). Follow‐up simple slopes analysis showed that at a higher level of childhood maltreatment (1 SD above the mean), eating disorder symptoms increased from discharge to 12‐month follow‐up; however, this effect was non‐significant (simple slope estimate [SE] = 0.12 [0.07], *t* = 1.56, *p* = 0.121). At lower levels of childhood maltreatment (1 SD below the mean), eating disorder symptoms significantly decreased from discharge to 12‐month follow‐up (simple slope estimate [SE] = −0.20 [0.07], *t* = −2.96, *p* = 0.003). In the model investigating childhood maltreatment and eating disorder symptoms in individuals with AN‐BP, there was no significant main effect for childhood maltreatment and no significant interactive effect.

**TABLE 2:** Descriptive statistics for eating disorder symptoms by timepoint.

	Discharge		3‐month		6‐month		12‐month	
	*M*	SD	*M*	SD	*M*	SD	*M*	SD
AN‐R								
Eating disorder symptoms	2.02	1.20	2.04	1.40	2.07	1.42	1.95	1.46
AN‐BP								
Eating disorder symptoms	2.86	1.60	3.10	1.50	2.95	1.61	2.89	1.60

Abbreviations: AN‐BP = Anorexia Nervosa binge‐eating/purging type; AN‐R = AN restricting type.

**TABLE 3:** Results of the multilevel models examining childhood maltreatment as a predictor of change in eating disorder symptoms from discharge to 12‐months post‐discharge.

	Estimate	SE	*t*	*p*	95% CI	
Model 1: AN‐R						
Intercept	−0.69	1.67	−0.41	0.680	−3.918	2.542
**Depressive symptoms**	**0.05**	**0.01**	**4.45**	**< 0.001**	**0.029**	**0.075**
Age^a^	−0.02	0.52	−0.04	0.971	−1.038	0.999
Comorbidity	−0.09	0.31	−0.30	0.769	−0.699	0.516
AN illness duration^a^	0.43	0.41	1.06	0.293	−0.369	1.238
Treatment duration	0.01	0.01	1.14	0.260	−0.005	0.018
**Time**	**−25.70**	**8.13**	**−3.16**	**0.002**	**−41.465**	**−9.945**
Childhood maltreatment^a^	0.10	0.39	0.27	0.788	−0.655	0.864
**Childhood maltreatment** ^a^ **× time**	**6.81**	**2.23**	**3.05**	**0.003**	**2.485**	**11.129**
Random effects	Estimate	SD				
Between‐person intercept	0.90	0.95				
Within‐person variance	0.45	0.67				
Model 2: AN‐BP						
Intercept	1.23	0.38	3.23	0.001	0.486	1.967
**Depressive symptoms**	**0.07**	**0.01**	**8.65**	**< 0.001**	**0.054**	**0.086**
Intensive treatment	0.10	0.23	0.41	0.684	−0.364	0.554
Time	−1.45	2.43	−0.60	0.551	−6.180	3.286
Childhood maltreatment^a^	< −0.01	0.01	−0.65	0.517	−0.019	0.009
Childhood maltreatment^a^ × time	0.02	0.05	0.42	0.677	−0.075	0.115
Random effects	Estimate	SD				
Between‐person intercept	0.78	0.88				
Within‐person variance	0.50	0.71				

*Note:* Bolded values are significant.

Abbreviations: AN‐BP = Anorexia Nervosa binge‐eating/purging type; AN‐R = AN restricting type; SE = standard error.

^a^ Log transformed variable was used.

The results of the multilevel modelling analyses investigating compulsivity and changes in eating disorder symptoms over the 12‐month period following discharge are presented in Table 4. In the model examining this in individuals with AN‐R, there was no significant main effect for compulsivity and no significant interactive effect. This same pattern of findings emerged in the model examining this in individuals with AN‐BP.

**TABLE 4:** Results of the multilevel models examining compulsivity as a predictor of change in eating disorder symptoms from discharge to 12‐months post‐discharge.

	Estimate	SE	*t*	*p*	95% CI	
Model 1: AN‐R						
Intercept	−0.75	1.38	−0.54	0.588	−3.416	1.921
**Depressive symptoms**	**0.05**	**0.01**	**4.51**	**< 0.001**	**0.029**	**0.074**
Age^a^	0.08	0.51	0.16	0.876	−0.927	1.088
Comorbidity	−0.10	0.31	−0.32	0.749	−0.708	0.509
AN illness duration^a^	0.47	0.40	1.19	0.239	−0.309	1.257
Treatment duration	0.01	0.01	1.14	0.258	−0.005	0.018
Time	−1.70	1.27	−1.34	0.182	−4.170	0.761
Compulsivity	0.02	0.05	0.31	0.754	−0.083	0.115
Compulsivity × time	0.31	0.35	0.89	0.372	−0.365	0.990
Random effects	Estimate	SD				
Between‐person intercept	0.93	0.97				
Within‐person variance	0.45	0.67				
Model 2: AN‐BP						
Intercept	0.96	0.25	3.85	< 0.001	0.475	1.450
**Depressive symptoms**	**0.06**	**0.01**	**7.51**	< **0.001**	**0.046**	**0.079**
Intensive treatment	0.08	0.23	0.35	0.727	−0.367	0.526
Time	−1.37	1.28	−1.07	0.284	−3.858	1.120
Compulsivity	0.06	0.04	1.65	0.103	−0.012	0.133
Compulsivity × time	0.16	0.25	0.63	0.527	−0.326	0.639
Random effects	Estimate	SD				
Between‐person intercept	0.77	0.88				
Within‐person variance	0.50	0.71				

*Note:* Bolded values are significant.

Abbreviations: AN‐BP = Anorexia Nervosa binge‐eating/purging type; AN‐R = AN restricting type; SE = standard error.

^a^ Log transformed variable was used.

## 4 Discussion

The purpose of this study was to investigate associations among childhood maltreatment, compulsivity, and eating disorder symptoms in individuals with AN. Consistent with our hypotheses, childhood maltreatment was associated with higher compulsivity at treatment admission but only in individuals with AN‐BP. Furthermore, childhood maltreatment moderated changes in eating disorder symptoms over the 12‐month period following discharge from treatment but only in individuals with AN‐R. In particular, individuals with AN‐R who had lower levels of maltreatment exhibited a significant decline in eating disorder symptoms from discharge to 12 months post‐discharge, whereas those with higher levels of maltreatment showed no change in eating disorder symptoms during this period. Contrary to our hypothesis, compulsivity at admission did not moderate changes in eating disorder symptoms from discharge to 12 months post‐discharge among individuals with AN‐R or AN‐BP. Overall, this study provides novel insights into the interplay between adverse childhood experiences, compulsivity, and eating disorder symptom trajectories in AN, highlighting distinct patterns for AN‐R and AN‐BP.

### 4.1 Childhood Maltreatment and Compulsivity in AN

More severe childhood maltreatment was associated with higher compulsivity at treatment admission among individuals with AN‐BP, supporting a link between childhood maltreatment and compulsivity in individuals with AN‐BP. In individuals with AN‐BP, compulsivity may originate as an adaptive response to an environment characterised by maltreatment, as repetitive, ritualised behaviour can enhance feelings of controllability and predictability (Tonna et al. 2019). Alternatively, childhood maltreatment may impair behavioural control, promoting impulsivity (i.e., the tendency to act on urges without foresight) that over time, evolves into compulsivity in individuals with AN‐BP (Kandeğer 2025). There is growing evidence that compulsivity and impulsivity are part of a single dimension, rather than distinct constructs, as both involve difficulties with behavioural control and may emerge at different stages within the same disorder (Kandeğer 2025). Moreover, childhood maltreatment has been shown to alter neural circuits underlying impulsive and compulsive behaviours (Kandeğer 2025; Malave et al. 2022). Research further indicates that impulsivity may serve as a pathway through which maltreatment (i.e., emotional abuse) contributes to eating disorder symptoms in individuals with AN (Barone et al. 2024). Future studies could explore whether individuals with AN‐BP who have histories of childhood maltreatment show a progression from impulsivity to compulsivity that is underpinned by difficulties with behavioural control. Overall, the present study represents a novel contribution to the existing literature by being the first study to our knowledge to demonstrate a link between childhood maltreatment and compulsivity among individuals with AN.

Contrary to expectations, childhood maltreatment was not associated with compulsivity among individuals with AN‐R. The differential findings for AN‐R and AN‐BP may relate to differences in the way compulsivity manifests in each subtype, and the ability of the repetition and automation subscale of the OBS‐SR to adequately capture this. This subscale primarily assesses behavioural manifestations of compulsivity, which may be more readily endorsed by individuals with AN‐BP, given the overt compulsive behaviours that characterise AN‐BP (i.e., binge eating and purging; American Psychiatric Association 2022; Dell’Osso et al. 2000; Dell'Osso et al. 2002). In contrast, individuals with AN‐R may endorse cognitive features of compulsivity, as AN‐R is often characterised by cognitive overcontrol or rigidity, which may not be adequately captured by the OBS‐SR (Buzzichelli et al. 2018). Future studies on childhood maltreatment and compulsivity in AN should consider utilising measures of compulsivity that capture cognitive and behavioural features of this construct. These contrasting findings for AN‐R and AN‐BP may also relate to differences in the timing of maltreatment between individuals with these disorders. Evidence suggests that maltreatment may be most likely to impair neurocognitive processes involved in the flexible regulation of behaviour if it occurs in early (as opposed to late) childhood (Cowell et al. 2015). While little is known about the timing of maltreatment among individuals with AN, individuals with AN‐BP are more likely than individuals with AN‐R to report that childhood abuse preceded the development of their eating disorder, which may suggest that they experience maltreatment earlier in childhood compared to individuals with AN‐R (Carter et al. 2006). Future research that assesses the developmental timing of maltreatment exposure may help to further clarify the association between childhood maltreatment and compulsivity in individuals with AN‐R and AN‐BP.

### 4.2 Childhood Maltreatment and the Trajectory of AN

Individuals with AN‐R who had lower levels of childhood maltreatment showed a significant reduction in eating disorder symptoms over the 12‐month period following discharge from intensive eating disorder treatment. In contrast, individuals with AN‐R who had higher levels of childhood maltreatment showed no significant change in eating disorder symptoms during this timeframe. These findings add to the literature suggesting that individuals with minimal or no history of maltreatment experience a more favourable course of eating disorder symptoms after discharge from treatment, compared to individuals with greater exposure to maltreatment (Convertino and Mendoza 2023; Day et al. 2024). There have been increasing calls in the eating disorder literature for more integrated models of care capable of addressing both eating disorder symptoms and symptoms related to trauma history (e.g., Day et al. 2024); findings from the present study suggest that childhood maltreatment may be a specific form of trauma history requiring additional relapse prevention efforts in the context of eating disorder treatment for AN‐R, regardless of whether or not trauma‐related symptoms are present.

Contrary to expectations and previous research showing that childhood maltreatment was more strongly associated with AN‐BP than AN‐R (Molendijk et al. 2017), childhood maltreatment at admission did not predict change in eating disorder symptoms from discharge to 12 months post‐discharge in individuals with AN‐BP in the current sample. This discrepancy may be due to differences in the design of our study compared to previous research, as our study assessed eating disorder symptom change over time following discharge from intensive treatment, while previous research assessed the severity of eating disorder symptoms at a single time point. Additionally, multiple longitudinal studies that demonstrated an association between childhood maltreatment and eating disorder symptoms in individuals with AN did not differentiate between AN subtypes (Cassioli et al. 2022; Eielsen et al. 2024), and one longitudinal study did not find a difference in the trajectory of eating disorder symptoms following intensive treatment between individuals with and without a history of childhood maltreatment (Calugi et al. 2018). Thus, while the present findings suggest that lower levels of maltreatment may predict a more favourable course of symptoms in AN‐R after treatment discharge, the association between childhood maltreatment and AN is complex and warrants further study. It is also possible that certain types of childhood maltreatment, rather than maltreatment in general, are relevant to the clinical course of AN‐BP, which was not captured in the current study. For instance, Monteleone et al. (2019) found that emotional abuse mediated relations between childhood maltreatment and eating disorder symptoms in individuals with bulimia nervosa and AN‐BP. Future research should explore whether specific types of childhood maltreatment, such as emotional abuse, are uniquely associated with post‐treatment trajectories of eating disorder symptoms in individuals with AN‐BP.

### 4.3 Compulsivity and the Trajectory of AN

Compulsivity at admission did not moderate changes in eating disorder symptoms from discharge to 12‐month post‐discharge in individuals with AN‐BP or AN‐R. Thus, while childhood maltreatment may be associated with elevated compulsivity in AN‐BP, compulsivity may not be relevant to the clinical course of eating disorder symptoms in AN‐BP following treatment discharge. Additionally, other correlates of childhood maltreatment may play a role in the post‐discharge trajectories of eating disorder symptoms in AN‐R. For example, childhood maltreatment is associated with difficulty adjusting emotion regulation strategies across changing circumstances (Pițur and Miu 2020), which is crucial for effective modulation of emotions (Bonanno and Burton 2013). Thus, future work could investigate whether the impact of childhood maltreatment on the ability to flexibly adapt emotion regulation strategies (rather than the ability to flexibly adapt behaviour more broadly) plays a role in maintaining AN‐R following intensive eating disorder treatment.

### 4.4 Strengths and Limitations

While this study has multiple strengths, including the clinical sample of individuals with AN and the longitudinal study design, it is not without limitations. First, childhood maltreatment was assessed using a retrospective self‐report measure, which is subject to recall bias that may lead to over‐ or under‐estimation of the effect of childhood maltreatment on eating disorder symptoms. Given that prospective and retrospective measures of childhood maltreatment often produce unique findings (Baldwin et al. 2019), reexamining the impact of childhood maltreatment on eating disorder symptom progression in youth with AN or youth at increased risk of AN using a prospective measure of childhood maltreatment is warranted. Second, compulsivity was only assessed at treatment admission. Therefore, we are unable to examine whether *changes* in compulsivity relate to changes in eating disorder symptoms following intensive eating disorder treatment among individuals with AN. Future research examining whether eating disorder symptoms are impacted by changes in compulsivity during treatment may elucidate whether compulsivity is a useful treatment target for individuals with AN. Third, this study focused on the trajectory of eating disorder symptoms following discharge from intensive eating disorder treatment; therefore, findings may not generalise to less intensive types of eating disorder treatment (e.g., outpatient treatment). Fourth, our study investigated the relation between childhood maltreatment and eating disorder symptoms, without consideration of how maltreatment relates to broader psychological symptoms, such as anxiety and depression. Given that childhood maltreatment (e.g., emotional abuse) has been linked to elevated symptoms of anxiety and depression (Monteleone et al. 2025), and these symptoms may negatively impact treatment outcomes for eating disorders (Gorrell et al. 2023), this topic warrants future investigation. Fifth, our sample was primarily comprised of individuals who were non‐Hispanic white females. It is critical that the relation between childhood maltreatment, compulsivity, and eating disorder symptoms be reassessed in a diverse group of individuals with AN prior to generalising the findings. Given limits in access to care for diverse groups and potential socioeconomic differences in childhood maltreatment rates (Lanier et al. 2014), community‐based research of individuals with AN who are not seeking intensive treatment may be warranted.

### 4.5 Conclusion

In summary, findings from this study replicate and extend previous research on the relations between childhood maltreatment, compulsivity, and AN psychopathology. Specifically, the current findings suggest that childhood maltreatment is associated with elevated compulsivity in individuals with AN‐BP. Additionally, individuals with AN‐R who report lower levels of childhood maltreatment may experience a more favourable course of eating disorder symptoms over the 12‐month following discharge from intensive eating disorder treatment, compared to those with higher levels of maltreatment. While further prospective research will be required to confirm these findings, they highlight childhood maltreatment as potentially a key factor to account for in the development of improved, personalised treatments for AN.

## Ethics Statement

All study procedures were approved by the University of Pittsburgh Institutional Review Board and conducted in accordance with the ethical standards of the 1964 Helsinki declaration and its later amendments.

## Consent

All participants provided written informed consent (or assent) prior to participating in the study.

## Conflicts of Interest

The authors declare no conflicts of interest.

## Supporting information


**Table S1**: Sociodemographic Characteristics of the Sample at Admission.

## Data Availability

Data are available from the corresponding author upon reasonable request.
