# Supplementary material for: Naturalistic Associations Between Childhood Maltreatment, Compulsivity, and Eating Disorder Symptoms Over a 12‐Months Period Among Individuals With Anorexia Nervosa
Source: Eur Eat Disord Rev. 2025 Oct 13;34(2):411–21. doi: 10.1002/erv.70041 (PMC12616517; doi:10.1002/erv.70041)
Supplement: Supplementary file 1 — Table S1: Sociodemographic Characteristics of the Sample at Admission. [file ERV-34-411-s001.docx]

| **Supplemental Table 1**  *Sociodemographic Characteristics of the Sample at Admission* | |
| --- | --- |
|  | *n* (%) or mean ± *SD* |
| Sex |  |
| Female | 185(95.4) |
| Male | 9(4.6) |
| Race/Ethnicity |  |
| Non-Hispanic white | 185(95.4) |
| Multi-ethnic | 4(2.1) |
| Asian | 2(1.0) |
| Hispanic | 2(1.0) |
| Black | 1(.5) |
| Age (years) | 26.50 ± 10.1 |
| Duration of illness (years) | 8.55 ± 9.0 |
| BMI at admission | 15.71 ± 1.8 |
| Level of care on admission  Inpatient  Day hospital | 162(83.5)  32(16.5) |
| Co-morbid psychiatric diagnosis |  |
| Yes | 162(83.5) |
| No | 32(16.5) |
| Education |  |
| Some high school | 40(20.6) |
| Completed high school | 27(13.9) |
| Trade school or technical school | 8(4.1) |
| Some college | 73(37.6) |
| Graduated from a 4-year college | 32(16.5) |
| Completed a post-graduate degree | 14(7.2) |
| *Note.* BMI = Body Mass Index (kg/m^2^) | |
